# Downregulating CD26/DPPIV by apigenin modulates the interplay between Akt and Snail/Slug signaling to restrain metastasis of lung cancer with multiple EGFR statuses

**DOI:** 10.1186/s13046-018-0869-1

**Published:** 2018-08-22

**Authors:** Jer-Hwa Chang, Chao-Wen Cheng, Yi-Chieh Yang, Wan-Shen Chen, Wen-Yueh Hung, Jyh-Ming Chow, Pai-Sheng Chen, Michael Hsiao, Wei-Jiunn Lee, Ming-Hsien Chien

**Affiliations:** 1 Department of Internal Medicine, School of Medicine, College of Medicine, Taipei Medical University, Taipei, Taiwan; 2 Division of Pulmonary Medicine, Department of Internal Medicine, Wan Fang Hospital, Taipei Medical University, Taipei, Taiwan; 3 Graduate Institute of Clinical Medicine, College of Medicine, Taipei Medical University, 250 Wu-Hsing Street, Taipei, 11031 Taiwan; 4 Genomics Research Center, Academia Sinica, Taipei, Taiwan; 5 Division of Hematology and Medical Oncology, Department of Internal Medicine, Wan Fang Hospital, Taipei Medical University, Taipei, Taiwan; 6 Institute of Basic Medical Sciences, College of Medicine, National Cheng Kung University, Tainan, Taiwan; 7 Department of Medical Laboratory Science and Biotechnology, College of Medicine, National Cheng Kung University, Tainan, Taiwan; 8 Department of Medical Education and Research, Wan Fang Hospital, Taipei Medical University, 111 Hsing Long Road, Section 3, Taipei, 11696 Taiwan; 9 Department of Urology, School of Medicine, Taipei Medical University, Taipei, Taiwan; 10 TMU Research Center of Cancer Translational Medicine, Taipei Medical University, Taipei, Taiwan

**Keywords:** Non-small cell lung cancer, Invasion, Metastasis, CD26/dipeptidyl peptidase IV, Akt, Snail, Slug, Apigenin

## Abstract

**Background:**

Metastasis rather than the primary cancer determines the survival of cancer patients. Activation of Akt plays a critical role in the epithelial-to-mesenchymal transition (EMT), the initial step in lung cancer metastasis. Apigenin (API), a flavonoid with a potent Akt-inhibitory effect, shows oncostatic activities in various cancers. However, the effects of API on metastasis of non-small cell lung cancer (NSCLC) remain unclear.

**Methods:**

NSCLC cell lines with different epidermal growth factor receptor (EGFR) statuses and in vivo orthotopic bioluminescent xenograft model were employed to determine antitumor activity of API. Western blot and genetic knockdown by shRNA or genetic overexpression by DNA plasmids were performed to explore the underlying mechanisms. The Cancer Genome Atlas (TCGA) database was used to investigate the prognosis of API-targeted genes.

**Results:**

API was demonstrated to inhibit the migration/invasion of NSCLC cells harboring different EGFR statuses via suppressing the Snail/Slug-mediated EMT. Mechanistic investigations showed that CD26/dipeptidyl peptidase IV (DPPIV) was downregulated by API following suppressive interplay of Akt and Snail/Slug signaling to modulate the EMT and the invasive ability of NSCLC cells. CD26 expression was positively correlated with the invasive abilities of NSCLC cells and a worse prognosis of lung cancer patients. Furthermore, we observed that patients with CD26^high^/Akt^high^ tumors had the shortest recurrence-free survival times. In vivo, API drastically reduced the growth and metastasis of A549 xenografts through targeting CD26.

**Conclusions:**

CD26 may be a useful biomarker for predicting NSCLC progression. API effectively suppressed lung cancer progression by targeting the CD26-Akt-Snail/Slug signaling pathway.

**Electronic supplementary material:**

The online version of this article (10.1186/s13046-018-0869-1) contains supplementary material, which is available to authorized users.

## Background

Lung cancer is the leading cause of cancer-related mortality worldwide [1], because most patients have already progressed to an advanced stage or have developed distant metastasis when diagnosed. More than 85% of lung cancers are histologically of non-small cell lung cancer (NSCLC) [2], and the prognosis of NSCLC patients with metastatic tumors or at stage IV is very poor, with only a median survival time of 8~ 10 months [3]. Currently, chemotherapy, radiotherapy, and targeted therapy are treatment options for advanced NSCLC. The response rate of standard first-line chemotherapy (platinum-based combined with third-generation cytotoxic agents) of advanced lung cancers has significantly improved survival times but with low short-term efficacy, high toxicity, and ultimately the development of drug resistance [4]. When chemotherapy is no longer effective, some targeted medicines such as gefitinib (an epidermal growth factor receptor (EGFR) tyrosine kinase inhibitor (TKI)) can be used for advanced NSCLC patients with EGFR mutations (deletions in exon 19 and L858R in exon 21) to further prolong patient survival [5]. However, most patients eventually have no effective response to gefitinib, because cancer cells develop a second mutation, such as T790 M in the *EGFR* gene [6]. Therefore, searching for new drugs with high efficacy and low toxicity is urgently needed.

Tumor metastasis is a continuous multi-step process, and the epithelial-to-mesenchymal transition (EMT) is one of the most important mechanisms in the initiation and promotion of tumor metastasis [7]. In NSCLC, the EMT of cells was reported to promote metastasis and also determine chemoresistance [8] and insensitivity to EGFR inhibitors [9]. The serine-threonine protein kinase, Akt, was reported to play a crucial role in NSCLC invasion [10], but the underlying molecular mechanisms of NSCLC invasion mediated by the phosphatidylinositol 3-kinase (PI3K)/Akt signaling pathway is not completely understood. At present, the EMT is known to be a cellular process subject to Akt kinase regulation. Activated Akt was shown to regulate several steps of the EMT, such as loss of cell-cell adhesion and polarization, morphological changes, induction of cell motility, and changes in the production of various proteins [11–13]. For example, Snail and Slug (Snail2), the most thoroughly investigated EMT regulators in lung cancer, are reportedly regulated by activated Akt [14]. PI3K/Akt can inhibit the degradation of Snail and Slug by targeting glycogen synthase kinase (GSK)-3β or by directly upregulating Snail expression in different cancer types [15–17]. Actually, the PI3K/Akt signaling pathway which mediates the EMT process has garnered widespread attention as a potential target for preventing and treating metastatic tumors. Therefore, investigating compounds with medicinal effects on Akt activation and the Snail family-mediated EMT should be a good strategy for NSCLC.

CD26, a 110-kDa type II transmembrane glycoprotein with dipeptidyl peptidase IV (DPPIV) activity in its extracellular domain, can cleave N-terminal dipeptides from polypeptides with an alanine or proline at the penultimate position [18]. Previously, CD26 was shown to participate in T-cell biology as a marker of T-cell activation or as a costimulatory molecule able to regulate signaling transduction pathways [19, 20]. Recently, CD26 was shown to play a critical role in cancer biology. For example, CD26 overexpression was associated with tumor aggressiveness in many cancer types such as astrocytomas [21], lymphomas [22], urothelial carcinoma [23], colorectal cancer [24], and gastrointestinal stromal tumors [25]. For example, CD26-positive colorectal cancer stem cells, which are mediators of the EMT, contribute to the invasive phenotype and metastatic capacity [24]. An in vivo study further showed that vildagliptin, a CD26 inhibitor, significantly suppressed metastasis of colorectal cancer [26]. These data emphasize the involvement of CD26 in cancer metastasis. So far, little information is known about the role of CD26 and its underlying mechanisms in regulating metastasis and invasion of NSCLC in vitro and in vivo.

Flavonoids are plentiful in fruits and vegetables and are a class of plant secondary metabolites with a ubiquitous phenolic structure. Recent cancer research studies have shown that flavonoids are highly promising compounds alone or in combination with other therapeutic agents against the growth and/or metastasis of different cancer cells in vitro and in vivo [27]*.* Apigenin (API), 4′,5,7-trihydroxyflavone, is one of the most common flavonoids and is abundantly present in various fruits, vegetables, and Chinese medicinal herbs [28]. API recently received much attention in cancer treatment, due to its potent anticancer activities in different cancer types in vitro and in vivo, including inducing apoptosis or autophagy, regulating the cell cycle, inhibiting migration/invasion, attenuating drug resistance, and stimulating immune responses [29]. The PI3K/Akt signaling pathway was reported to play a critical role during most anticancer processes induced by API [29].

Although API was recently shown to inhibit proliferation by targeting Akt in NSCLC cells harboring the wild-type EGFR [30], whether API has a broad impact on NSCLC with different EGFR mutation statuses, how API impacts the metastatic ability of NSCLC cells in vivo, and what the underlying mechanisms of API are in regulating Akt activity and modulating cell motility are still undefined. In the present study, we further investigated the antimetastatic effect of API on four NSCLC cell lines which harbor the wild-type (WT) or mutant EGFR and defined its underlying mechanisms in vitro and in an orthotopic xenograft model.

## Methods

### Materials

API (A3145) and dimethyl sulfoxide (DMSO) were purchased from Sigma-Aldrich (St. Louis, MO). Fetal bovine serum (FBS), antibiotics, molecular weight standards, trypsin-EDTA, trypan blue stain, and all medium additives were obtained from Life Technologies (Gaithersburg, MD). An enhanced chemiluminescence kit was purchased from Amersham (Arlington Heights, IL). An anti-matrix metalloproteinase (MMP)-3 antibody was purchased from Epitomic (Burlington, CA). Antibodies specific for fibronectin and MMP-9 were obtained from Abcam (Cambridge, MA). Antibodies specific for CD26, MMP-2, Snail, Slug, Twist, phosphatase and tensin homolog (PTEN), and unphosphorylated and phosphorylated (p-) forms of the corresponding Akt and epidermal growth factor receptor (EGFR) were obtained from Cell Signaling Technology (Danvers, MA). Antibodies specific for vimentin and N-cadherin were purchased from BD Biosciences (San Jose, CA). Antibodies specific for presenilin-1 and β-actin were obtained from Santa Cruz Biotechnology (Santa Cruz, CA). Polyvinylidene fluoride (PVDF) membranes for Western blotting were purchased from Bio-Rad (Hercules, CA). Unless otherwise specified, other chemicals used in this study were purchased from Sigma Chemical (St. Louis, MO).

### Cell lines and cell culture

The A549, H1975, and HCC827 NSCLC cell lines were purchased from American Type Culture Collection (ATCC, Manassas, VA), and a series of NSCLC cell lines, CL1–0, CL1–3, and CL1–5, in ascending order of invasiveness, were established in the National Health Research Institute laboratory [31]. PC9 cells were developed by Lee and colleagues at National Cancer Center Hospital (Tokyo, Japan) [32]. All cells were maintained in RPMI 1640 supplemented with 10% FBS and 1% penicillin-streptomycin-glutamine. All cells were incubated at 37 °C in a humidified 5% CO_2_ atmosphere.

### Cell viability assay (MTS assay)

A549, CL1–5, HCC827, and H1975 NSCLC cells (5 × 10^3^) were seeded in 96-well plates, treated with various concentrations of API (5~ 80 μM) for 24 and 48 h, and then subjected to a cell-viability assay (MTS assay; Promega, Madison WI) according to the manufacturer’s instructions. Data were collected from three replicates.

### Plate clonogenic assay

NSCLC cells were diluted and seeded at 1000 cells/well in six-well plates and incubated for 24 h. Subsequently, various concentrations (5~ 40 μM) of API were added for 24 h, and then continuously incubated in new fresh medium at 37 °C. After incubation for 7~ 10 days, cells were stained with crystal violet, and a colony was defined as consisting of more than 50 cells.

### Transwell migration and invasion assays

Migration and invasion assays were performed according to our previous study [33]. Briefly, 3 × 10^4^ cells were plated in a uncoated top chamber (24-well insert; pore size, 8 μm; Corning Costar, Corning, NY) for the transwell migration assay. The invasion assay used 4 × 10^4^ cells (A549 and HCC827) or 3 × 10^4^ cells (CL1–5 and H1975) plated in a Matrigel (BD Biosciences, Bedford, MA)-coated top chamber. In both assays, cells which had been pretreated for 24 h with API (5~ 40 μM) were plated in medium without serum or growth factors, and medium supplemented with 10% serum was used as a chemoattractant in the lower chamber. Cells that were allowed to migrate or invade for 24 h were fixed with methanol and stained with crystal violet. The number of cells migrating through or invading through the membrane was counted under a light microscope (× 100 or × 200, three random fields per well).

### Immunofluorescence (IF) microscopy

IF techniques were used to observe actin rearrangement after treatment of NSCLC cells with API or the vehicle. Cells were grown on coverslips and fixed in 4% paraformaldehyde, permeabilized with 0.1% Triton X-100, and stained with Alexa Fluor 594 Phalloidin (Thermo Fisher Scientific, Rockford, IL) in the dark. Slides were examined and photographed using a Zeiss Axiophot fluorescence microscope (Carl Zeiss Microimaging, Gottingen, Germany). Nuclei were counterstained with 4′,6-diamino-2-phenylindole (DAPI).

### Preparation of total cell extracts and western blot analysis

Protein lysates were prepared as described previously [34]. A Western blot analysis was performed with indicated primary antibodies and horseradish peroxidase-conjugated secondary antibodies. After washing, blots were incubated with the Western blotting reagent ECL (TOOLS, New Taipei City, Taiwan), and chemiluminescence was detected by the chemiluminescence imaging system, MultiGel-21 (TOP BIO, New Taipei City, Taiwan). Furthermore, the same blots were stripped by stripping buffer (TOOLS, New Taipei City, Taiwan) and reprobed with the β-actin antibody as an internal control.

#### Transient transfection of DNA and small interfering (si)RNA

siRNA for human CD26 (s4245) was obtained from Thermo Fisher Scientific. The pENTER-CD26 plasmid was obtained from Vigene Biosciences (Rockville, MD), and the myr-Akt, pLEX-Snail, and pCIneo-Slug plasmids were obtained from Dr. C.C. Chen and Dr. T.C. Kuo (National Taiwan University, Taipei, Taiwan). To knock down CD26 or overexpress CD26, Akt, Snail, or Slug, semiconfluent cultures of NSCLC cells in a 6-mm^2^ Petri dish were transfected with 50 nM of siRNA using GenMute™ siRNA Transfection Reagent (SignaGen Laboratories, Gaithersburg, MD) or 3 μg of an empty or expression vector using Lipofectamine 3000 Transfection Reagent (Invitrogen, Carlsbad, CA) for 6 h according to the manufacturer’s instructions. At 24 h after transfection, cells were analyzed for invasion/migration and expressions of CD26, Snail, Slug, and p-Akt.

### Lentiviral production and infection

Short hairpin (sh) RNAs were purchased from the National RNAi Core Facility at Academic Sinica (Taipei, Taiwan). The target sequence of CD26 shRNA was 5’-ACACTCTAACTGATTACTTAA-3. The shRNA lentivirus was produced as previously described [35].

### RNA preparation and reverse-transcriptase polymerase chain reaction (RT-PCR)

Messenger (m) RNA was isolated and amplified as described previously [35]. Primer sequences of CD26 were F: 5’-GAATGCCAGGAGGAAGGAATCT-3′ and R: 5’-TATTCCACACTTGAACACGCCA-3′.

### In vivo lung cancer orthotopic model

All animal experiments were performed under a protocol approved by the Institutional Animal Care and Use Committee of Taipei Medical University. For the CD26 overexpression and knockdown experiments in an orthotopic xenograft model, 5-week-old nonobese diabetic (NOD)-SCID male mice were anesthetized with isoflurane and placed in the right lateral decubitus position; then the A549-mock-luciferase, A549-CD26-luciferase, or A549-sh-CD26-luciferase (sh-775 and sh-777) stable cell lines (10^6^ cells) were resuspended in a 1:1 mixture of phosphate-buffered saline (PBS) and GFR-Matrigel and injected into the left lung parenchyma of a NOD-SCID mouse using a 30-gauge needle. After 7 days, the mice were randomized into six groups according to bioluminescence images taken using the Xenogen IVIS-Spectrum system (Caliper; Xenogen, CA), and treatment was initiated according to similar mean tumor sizes in each group. Subsequently, mice were intraperitoneally (IP) administered 3 mg/kg API or the vehicle (10% DMSO in PBS) 5 days/week. The day after API treatment, the mice were injected with D-luciferin and imaged for 1~5 min using this live imaging device to monitor the tumor size and location in real time. After 28 days, A549-injected mice were sacrificed, and luciferase activities in the excised lungs were further determined using the In Vivo Imaging System (IVIS)-Spectrum system. Mouse lungs were also fixed, sectioned, and stained with hematoxylin and eosin (H&E).

### Statistical analysis

Values are presented as the mean ± standard deviation (SD). The statistical analysis was performed using Statistical Package for Social Science software, vers. 16 (SPSS, Chicago, IL). Data were analyzed using Student’s *t*-test when two groups were compared. A one-way analysis of variance (ANOVA) followed by Tukey’s post-hoc test was used to analyze three or more groups. Statistical analyses of the correlation between CD26 and the invasiveness of NSCLC cells were performed using Spearman rank correlations. Differences were considered significant at a 95% confidence interval (*p* < 0.05).

## Results

### API suppresses migration, invasion, and colony formation of human NSCLC cells harboring different EGFR statuses

**Fig. 1:**
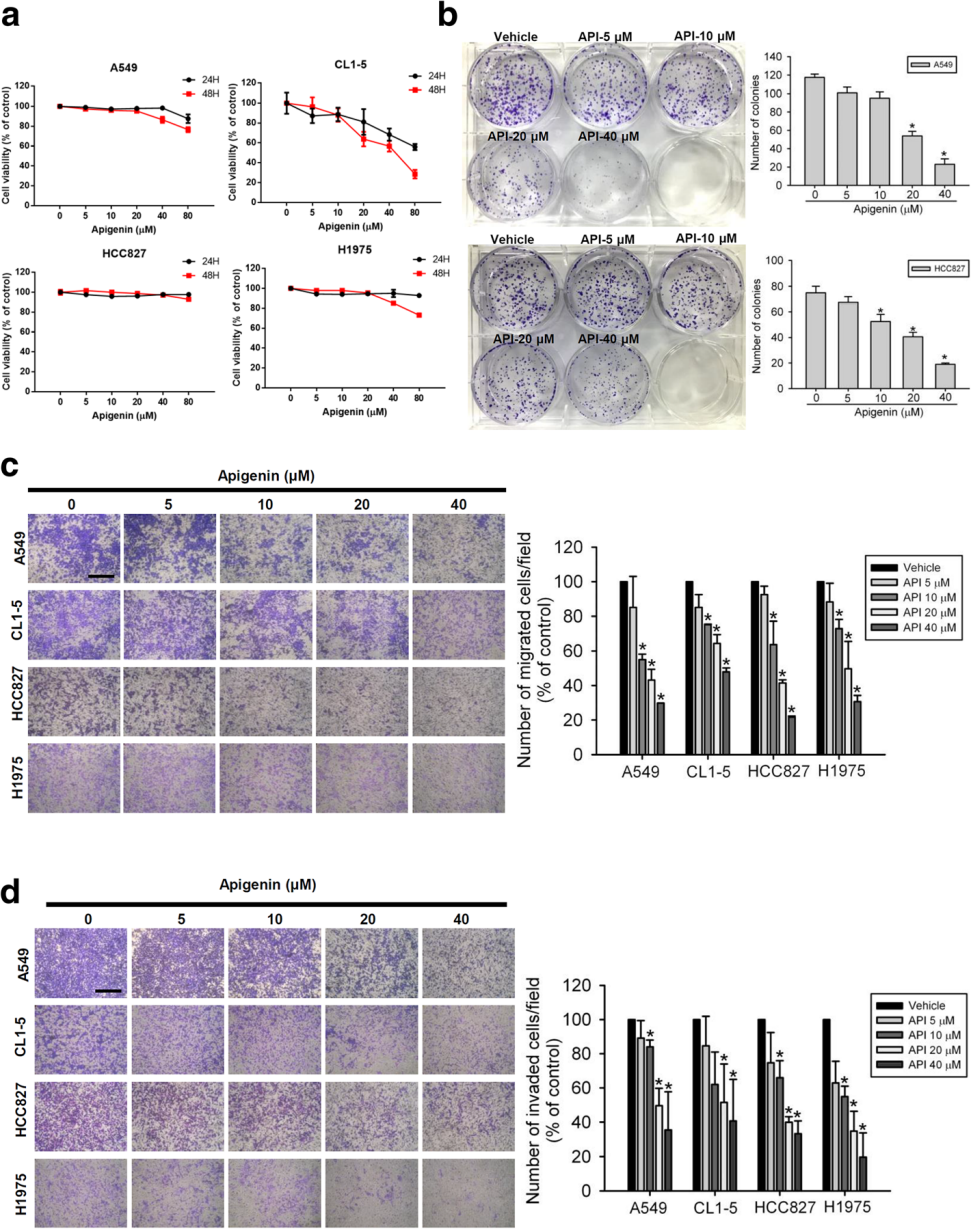
Apigenin (API) inhibits colony formation, cell migratory, and invasive abilities of non-small cell lung cancer (NSCLC) cells harboring different epidermal growth factor receptor (EGFR) statuses. **a** Four NSCLC cell lines, A549 and CL1–5 (wild-type EGFR), HCC827 (del E746-A750), and H1975 (L858R/T790 M), were treated with the indicated concentrations of API for 24 or 48 h, and cell viability was determined with an MTS assay. **b** A549 and HCC827 cells were treated with the vehicle or API (5~40 μM) for 24 h; then, the death-inducing effects of API on cells were determined by counting the colonies formed. **c, d** Four NSCLC cell lines (A549, CL1–5, HCC827, and H1975) were treated with indicated concentrations of API for the transwell-migration (**c**) and Matrigel-invasion (**d**) assays. **b-d** Left: Representative photomicrographs (100×). Scare bar, 500 μm. Right: Data are presented as the mean ± SD of at three independent experiments. * *p* < 0.05, compared to the vehicle group

Recent reports indicated that API can inhibit the in vitro proliferation and motility of human A549 NSCLC cells which harbor the WT EGFR [30]. To further investigate the broad therapeutic potential of API against NSCLC, we examined the effects of API on cell migration and invasion of NSCLC cells harboring different EGFR statuses including the exon 19 deletion (del E746-A750) and L858R/T790 M. First of all, four NSCLC cell lines including A549 and CL1–5 (WT EGFR), HCC827 (del E746-A750), and H1975 (L858R/T790 M) were treated with various concentrations (0~ 80 μM) of API for 24 or 48 h to determine the cytotoxic effects of API. An MTS analysis indicated that only at the highest concentration of 80 μM, API partially suppressed or did not alter the viability of these NSCC cell lines after 24 h of treatment, compared to that of the controls (Fig. 1a). In contrast to short-term treatment (24 h), the colony-formation assay showed that long-term treatment (7 days) with API significantly inhibited the colony-forming abilities of A549, CL1–5, HCC 827, and H1975 cells even at lower concentrations of 10~20 μM (Fig. 1b, Additional file 1: Figure S1). These results indicated that 24-h treatment with API (0~ 40 μM) exhibited very low cytotoxicity in all tested NSCLC cells, but long-term treatment with API efficiently inhibited the in vitro tumorigenicity of these cells. To further determine the effects of short-term API treatment on the cell motility of NSCLC cells, transwell-migration and Matrigel-invasion assays were performed. As shown in Fig. 1c and d, after 24 h of treatment of A549, CL1–5, HCC827, and H1975 cells with API (0~ 40 μM), the migratory and invasive abilities of these cells were all concentration-dependently suppressed, suggesting that even non-toxic concentrations of API can abolish the cell motility of these NSCLC cells.

### API inhibits the snail family-regulated EMT and invasive ability of NSCLC

The EMT and extracellular matrix (ECM) degradation are two key steps in cancer metastasis and were also shown to play critical roles in regulating the invasion and migration of NSCLC [36, 37]. Previous studies indicated that actin remodeling is an important upstream regulator of the EMT in metastatic cancer cells [38].

**Fig. 2:**
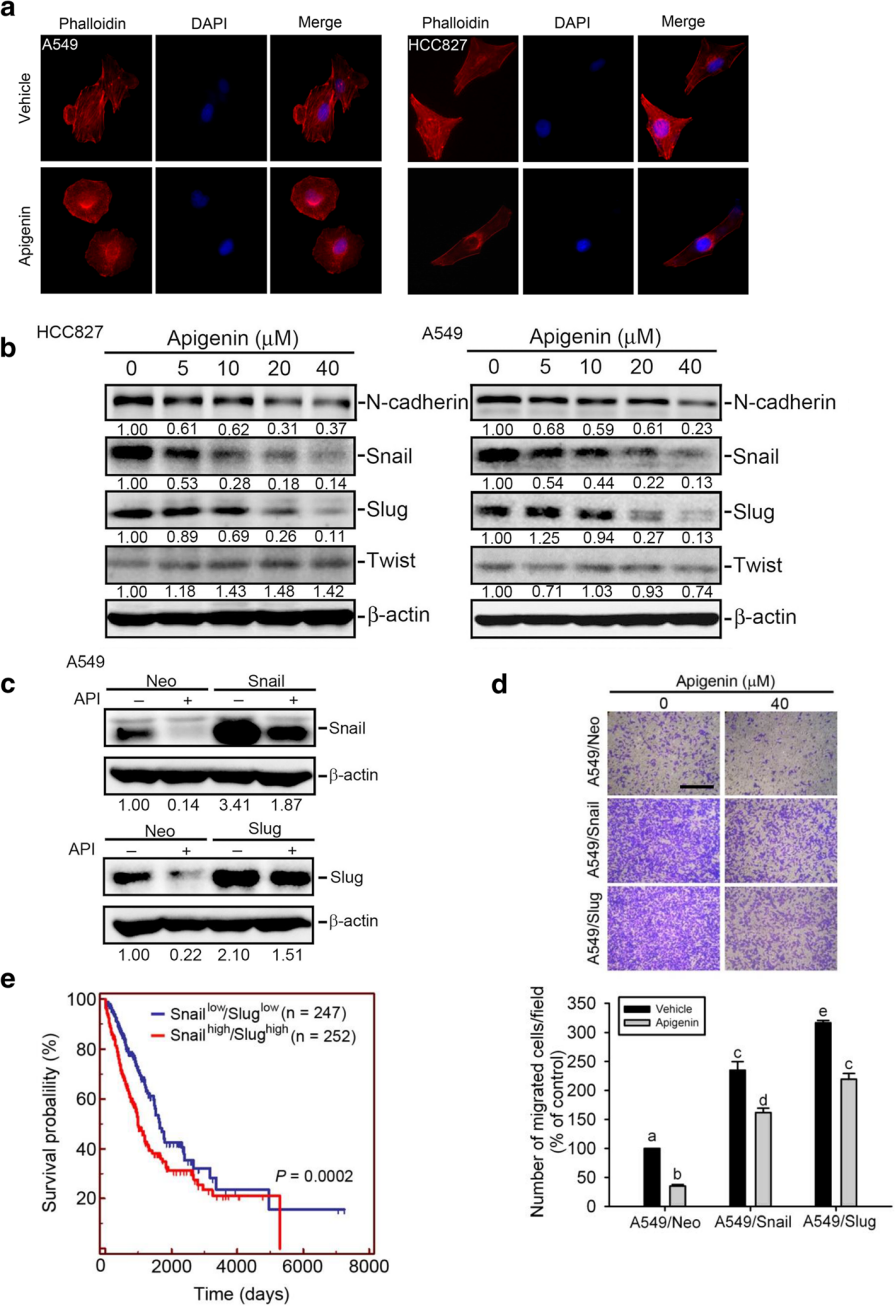
Apigenin (API) regulates cellular microfilament bundle and epithelial-to-mesenchymal transition (EMT)-related regulators in non-small cell lung cancer (NSCLC) cells. **a** Cellular microfilament bundle rearrangements were induced by treating A549 and HCC827 NSCLC cells with API. Cells were treated with 40 μM of vehicle or API for 24 h. Cells were fixed and stained for F-actin by Alexa Fluor 594 Phalloidin (red). Nuclei were counterstained with DAPI (blue). Original magnification, 400×. **b** A mesenchymal marker, N-cadherin, and the EMT-related transcriptional factors, Snail and Slug, were downregulated in A549 and HCC827 NSCLC cells after 24 h of treatment with API (0~ 40 μM). Quantitative results of indicated protein levels, which were adjusted to the β-actin protein level. **c, d** Snail family members play a critical role in API-mediated inhibition of the cell migratory ability. Western blot analysis of Snail (**c**, upper panel) and Slug (**c**, lower panel) in A549 cells which were transfected with either pLEX-Snail (A549/Snail), pCIneo-Slug (A549/Slug), or their respective controls (A549/Neo) followed by API or vehicle treatment for an additional 24 h. Quantitative results of Snail family protein levels, which were adjusted to the β-actin protein level. Migratory abilities of A549/Snail, A549/Slug, and A549/Neo which were treated with the vehicle or 40 μM API (**d**). Quantitative results by counting migrated cells in a 100× field. Multiples of differences are presented as the mean ± SD of three independent experiments. Data were analyzed with a one-way ANOVA and Tukey’s post-hoc tests at 95% confidence intervals; different letters represent different levels of significance. Scare bar, 500 μm. **e** Combined expressions of high Snail and high Slug proteins were correlated with the worst overall survival of patients with lung cancer. The *p* value refers to a comparison between Snail^high^/Slug^high^ and Snail^low^/Slug^low^. The lung cancer dataset was retrieved from TCGA

To determine whether API suppresses cell motility accompanied by changes in the actin cytoskeleton, Alexa Fluor 594 phalloidin and DAPI were used to respectively stain actin filaments and nuclei. As shown in Fig. 2a, A549 and HCC827 control cells displayed well-organized F-actin-containing microfilament bundles within the cytoplasm, whereas API-treated cells contained few microfilament bundles, indicating that F-actin-containing microfilament bundle rearrangements may be involved in API-modulated cell motility. Furthermore, we determined the effects of API on the EMT progression of NSCLC cells. In A549 and HCC827 cells, expressions of mesenchymal marker, N-cadherin, and the EMT-promoting transcription factors, Snail and Slug, were all concentration-dependently downregulated after treating cells with API (0~ 40 μM) for 24 h (Fig. 2b). These data suggested that downregulating Snail family members (Snail and Slug) might be critical for the API-mediated suppression of the EMT in EGFR WT and EGFR mutant NSCLC cells. Next, to determine whether Snail family members play critical roles in API-modulated cell motility, we overexpressed Snail or Slug in A549 cells to reverse the inhibitory effect of API on the Snail family (Fig. 2c). Meanwhile, the migratory ability was promoted in Snail- or Slug-overexpressing A549 cells compared to control A549 cells (A549/Neo), and the API-mediated suppression of the migratory ability in A549 cells was respectively reversed when Snail or Slug was overexpressed in cells (Fig. 2d). To elucidate the clinical relevance of Snail family members, a cohort of 499 lung cancer cases from The Cancer Genome Atlas (TCGA) was analyzed. Results from a Kaplan-Meier plot showed that patients with tumors exhibiting high expression levels of both Snail and Slug had significantly shorter survival times compared to patients with tumors exhibiting low expression levels of Snail and Slug (Fig. 2e). These data suggest that downregulating Snail and Slug is crucial for the API-mediated inhibition of EMT progression and cell invasion, and those high levels of Snail and Slug predicted a poor prognosis in patients with NSCLC.

### An interplay between Akt activation and expressions of snail family members is involved in API-mediated inhibition of cell motility

**Fig. 3:**
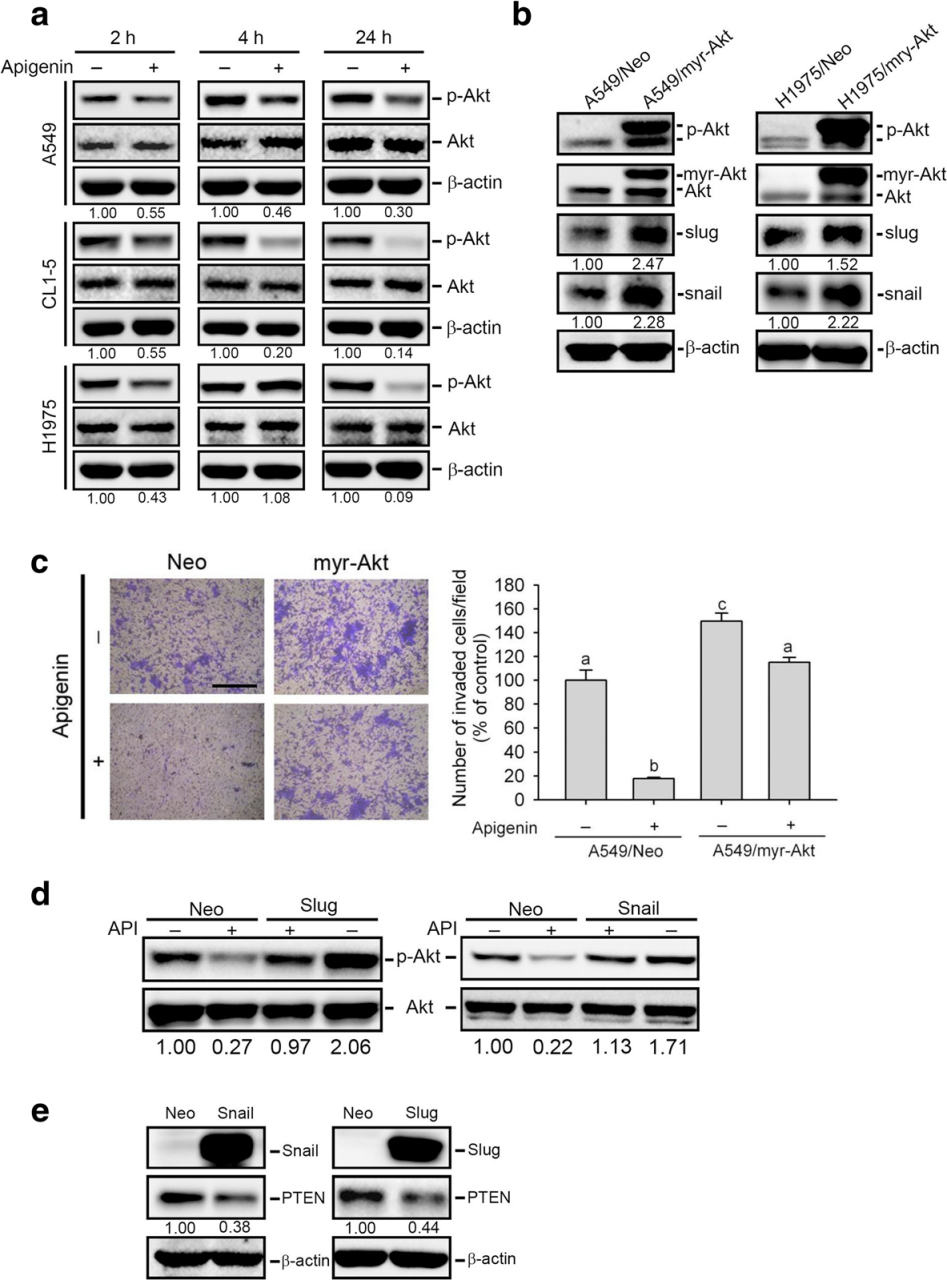
Interplay between Akt activation and Snail family expression is involved in apigenin (API)-mediated inhibition of cell motility. **a** Akt activation (phosphorylation) was assessed by a Western blot analysis in three non-small cell lung cancer (NSCLC) cell lines (A549, CL1–5, and H1975) after treatment with API (40 μM) for 2, 6, and 24 h. **b, c** Western blot analysis of p-Akt, Snail, and Slug expressions (**b**) and the invasive ability (**c**) of NSCLC cells (A549 and H1975) which were transiently transfected with a vector control or myr-Akt followed by API (40 μM) or vehicle treatment for an additional 24 h. Quantitative results by counting invaded cells in a 100× field. Multiples of differences are presented as the mean ± SD of three independent experiments. Data were analyzed with a one-way ANOVA and Tukey’s post-hoc tests at 95% confidence intervals; different letters represent different levels of significance. Scare bar, 500 μm. **d** Western blot analysis of Akt phosphorylation in A549 cells which were transiently transfected with either pLEX-Snail (A549/Snail), pCIneo-Slug (A549/Slug), or their respective controls (A549/Neo) followed by API (40 μM) or vehicle treatment for an additional 24 h. **e** Effect of Snail or Slug overexpression on phosphatase and tensin homolog (PTEN) expression in A549 cells. Quantitative results of p-Akt and other indicated proteins were respectively adjusted to total Akt protein and β-actin protein levels

Previous studies indicated that the Akt signaling pathway plays a crucial role in most API-mediated anticancer processes and increases the stability and transcription of Snail family members during the EMT of cancer cells [15–17, 29]. Herein, we found that API inhibited activation of Akt at indicated time points (2, 4, and 24 h) in A549, CL1–5, and H1975 cells (Fig. 3a). Moreover, we further overexpressed the constitutively activated Akt, myr-Akt, in A549 and H1975 cells and found that overexpressing myr-Akt significantly increased Snail and Slug expression levels in both cells (Fig. 3b). Functionally, we observed that overexpressing activated Akt significantly reversed API-induced inhibition of invasion in A549 cells (Fig. 3c). These data suggest that API suppression of Snail family-mediated cell motility is attributable to its capacity to inhibit Akt activation. In contrast to Akt-mediated Snail and Slug expressions, Snail was also reported to induce the EMT through activating the Akt pathway in NSCLC [39]. Our results showed that overexpression of Snail and Slug could reverse the API-mediated suppression of Akt activation (Fig. 3d). Moreover, overexpression of Snail and Slug also inhibited expression of the PI3K/Akt pathway suppressor, PTEN (Fig. 3e), suggesting that API may inhibit Snail family members to maintain PTEN expression and further suppress the PI3K/Akt pathway. Taken together, these data suggest that a regulatory network between Akt activation and Snail family expressions exists in API-mediated inhibition of cell motility.

### Alterations of protease levels in API-treated NSCLC cells

**Fig. 4:**
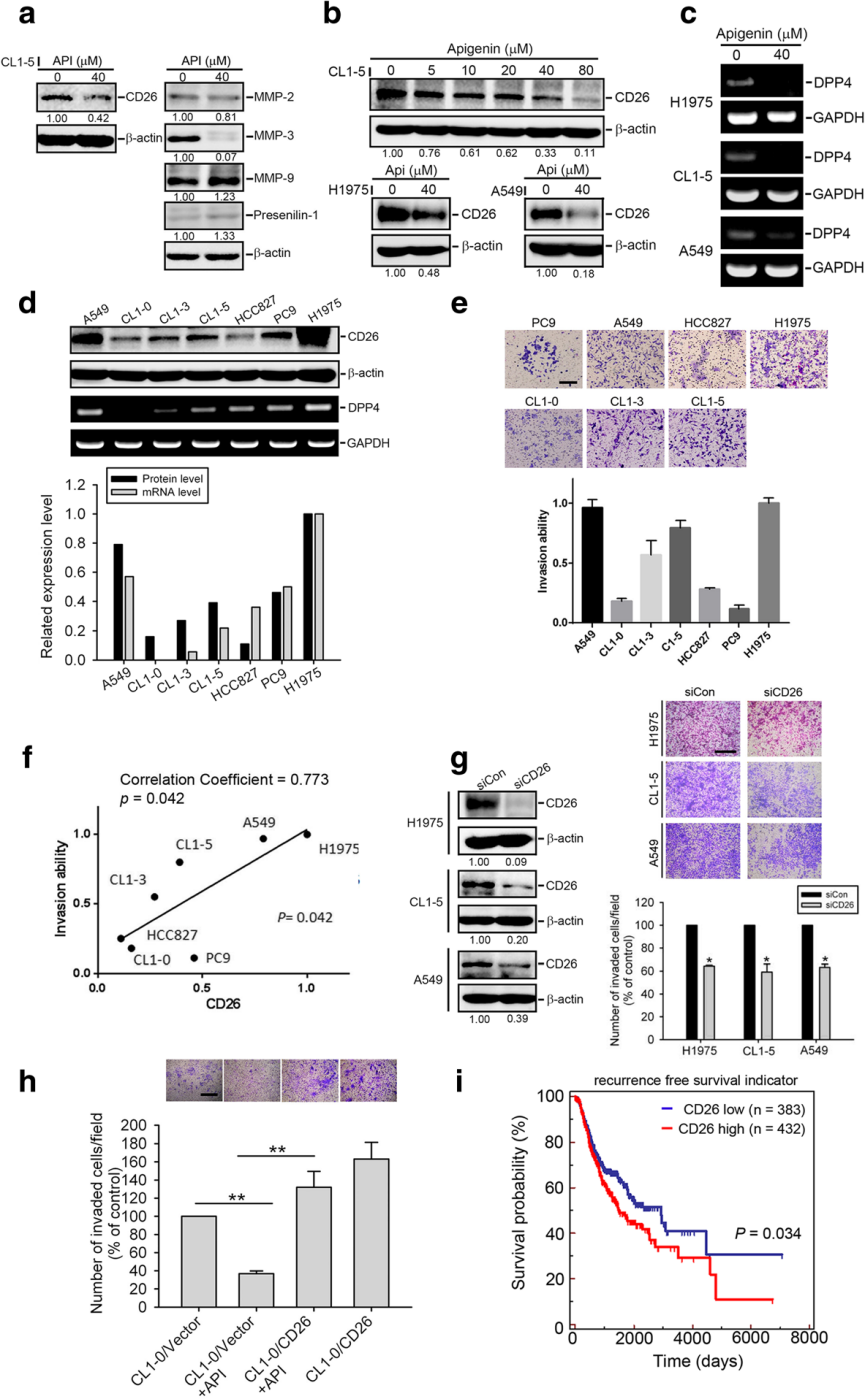
CD26 is important for apigenin (API)-modulated motility and positively correlates with invasiveness of non-small cell lung cancer (NSCLC) cells. **a** Changes in expressions of different proteases in CL1–5 cells following 24 h of treatment with API. **b** CL1–5 cells (upper panel) were treated with various concentrations of API or H1975, and A549 cells (lower panel) were treated with 40 μM API for 24 h, after which a Western blot analysis was performed. Quantitative results of indicated proteins were adjusted to β-actin protein levels. **c** NSCLC cells were treated with 40 μM API for 24 h and then subjected to an RT-PCR to analyze CD26 mRNA expression. **d** Upper panel, protein and mRNA levels of CD26 in NSCLC cell lines. Lower panel, quantitative results of CD26 protein and mRNA levels which were respectively normalized to β-actin and GAPDH levels. **e** In vitro invasive abilities of NSCLC cell lines. Quantitative results by counting invaded cells in a 200× field. Values are presented as the mean ± SD of three independent experiments. Scare bar, 200 μm. **f** Correlations between CD26 protein expression levels and the invasiveness of NSCLC cell lines. Spearman correlation coefficient = 0.773; *p* = 0.042. **g** A549, CL1–5, and H1975 cells were transiently transfected with CD26-specific siRNA or control siRNA and subjected to invasion assays. Left panel, Western blot analysis of CD26 expression. Right panel, CD26-specific siRNA suppresses the invasive abilities of NSCLC cells. Quantitative results by counting invaded cells in a 100× field. Multiples of differences are presented as the mean ± SD of three independent experiments. * *p* < 0.05, compared to the control group. Scare bar, 500 μm. **h** Invasive ability in CL1–0 cells which were transiently transfected with a vector control (CL1–0/Neo) or pENTER-CD26 (CL1–0/CD26) followed by API or vehicle treatment for an additional 24 h. Quantitative results by counting invaded cells in a 100× field. Values are presented as the mean ± SD of three independent experiments. ** *p* < 0.01, compared to the vehicle groups. Scare bar, 500 μm. **i** Kaplan-Meier analysis of *CD26* gene expression in lung cancer tissues. The lung cancer dataset was retrieved from TCGA

Recent findings indicated that several proteases, including MMP-2, MMP-3, MMP-9, presenilin 1, and CD26, are promoters and mediators of EMT processes in different cancer types [24, 40, 41]. We next screened the effect of API on these proteases and found that MMP-3 and CD26 were considerably downregulated in CL1–5 cells after API treatment (Fig. 4a). In a further check of the inhibitory effect of API on proteases in other NSCLC cell lines (A549 and H1975), Western blot data showed that only CD26 was consistently suppressed by API in all tested NSCLC cells (Fig. 4b, Additional file 1: Figure S2). Moreover, results showed that API concentration-dependently suppressed CD26 expression at concentrations of 10~ 80 μM in CL1–5 cells (Fig. 4b). In addition to the protein level, CD26 mRNA expression was also inhibited by API in A549, CL1–5, and H1975 cells (Fig. 4c). These data suggest that inhibition of CD26 by API may be a general phenomenon in NSCLC cells. To further determine the functional role of CD26 in NSCLC, we investigated the correlation between the CD26 expression and the cell invasive ability. We first detected expression levels of CD26 in a set of NSCLC cell lines (A549, CL1–0, CL1–3, CL1–5, HCC827, PC9, and H1975) using Western blotting and an RT-PCR (Fig. 4d). Next, the invasive abilities of these cell lines were further evaluated, and we found a variety of invasive abilities, with A549 and H1975 cells exhibiting relatively high invasive abilities (Fig. 4e). Moreover, we observed that H1975 and A549 cells also expressed relatively high CD 26 protein and mRNA levels among these cell lines (Fig. 4d). We further combined results from Fig. 4d and e and found a significant correlation (correlation coefficient *r* = 0.773, *p* = 0.042) between CD26 protein levels and the invasive abilities of these cell lines (Fig. 4f). Next, to determine whether CD26 modulates the cell invasive ability, we knocked-down CD26 in A549, H1975, and CL1–5 cells. The knockdown efficiency of CD26-specific siRNA was detected by a Western blot analysis (Fig. 4g, left panel). After knocking-down CD26 in these cells, we found their invasive abilities were all significantly attenuated compared to control cells (Fig. 4g, right panel). In comparison, overexpression of CD26 significantly increased the cell-invasive abilities of poorly invasive CL1–0 cells and also reversed the API-mediated suppression of the invasive ability of CL1–0 cells (Fig. 4h). In the clinic, we analyzed CD26 gene expression data obtained from TCGA and found that CD26 expression was inversely correlated with recurrence-free survival of lung cancer patients (Fig. 4i). These results suggested that CD26 may play a critical role in the API-regulated invasive ability of NSCLC cells and may be associated with the poor prognosis of patients with lung cancer.

### CD26 is an upstream regulator involved in API-regulated Akt activation and the EMT in NSCLC cells

**Fig. 5:**
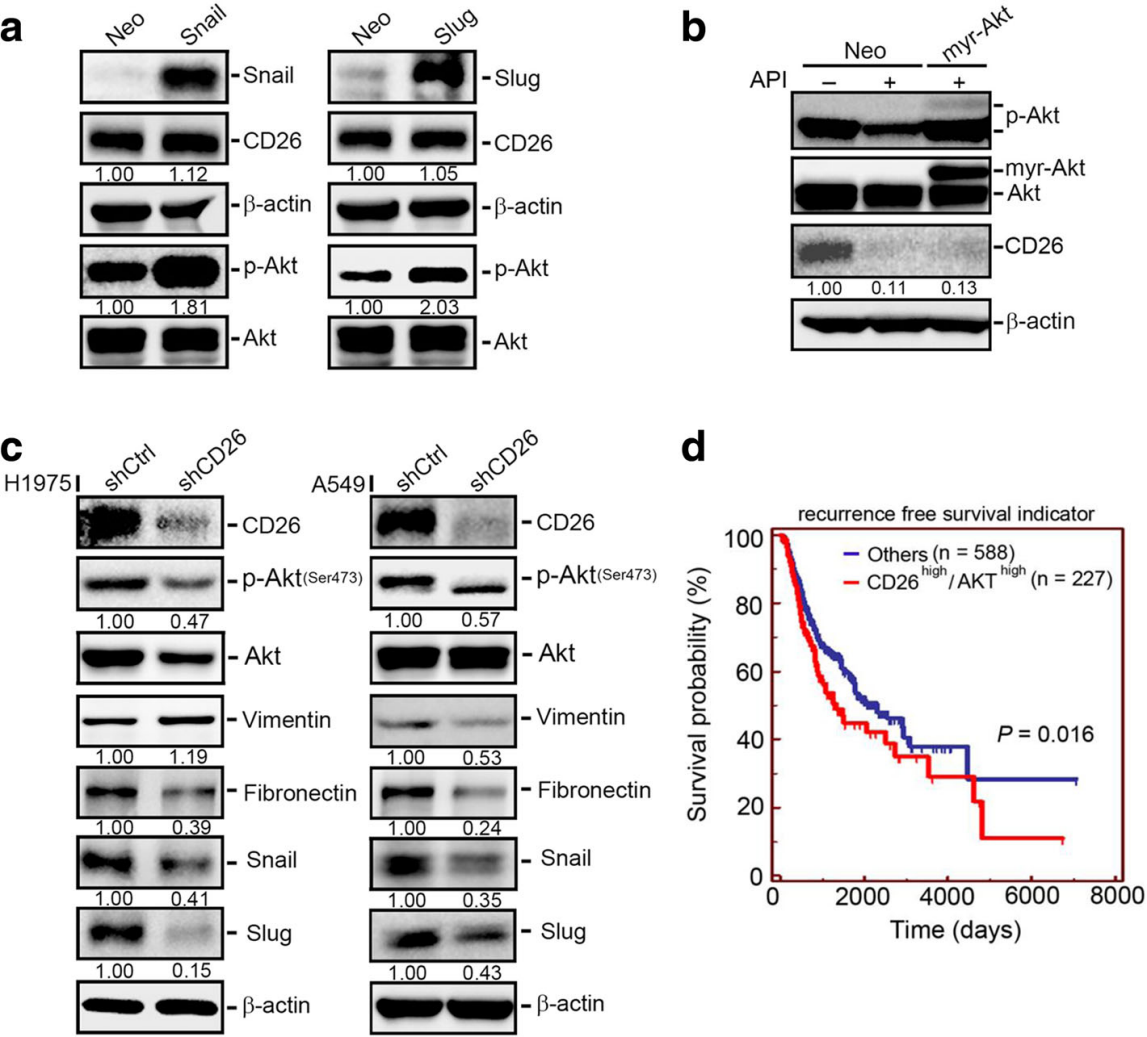
CD26 is an upstream regulator involved in apigenin (API)-regulated Akt activation and the epithelial-to-mesenchymal transition (EMT) in non-small cell lung cancer (NSCLC) cells. **a** Western blot analysis of CD26 expressions in A549 cells overexpressing Snail (left panel) and Slug (right panel). **b** Western blot analysis of CD26 expressions in A549 cells which were transiently transfected with a vector control (A549/Neo) or myr-Akt (A549/myr-Akt) followed by API or vehicle treatment for an additional 24 h. **c** A549 and H1975 cells were infected with a lentivirus carrying CD26 shRNA or shGFP (shCtrl) and subjected to a Western blot analysis to determine expressions of CD26, p-Akt, and EMT-related regulators (vimentin, fibronectin, Snail, and Slug). Quantitative results of p-Akt and other indicated proteins were respectively adjusted to total Akt protein and β-actin protein levels. **d** Combined expressions of high CD26 and high Akt proteins correlated with the recurrence-free survival of patients with lung carcinoma. The *p* value refers to a comparison between CD26^high^/Akt^high^ and other groups (CD26^high^/Akt^low^, CD26^low^/Akt^high^, or CD26^low^/Akt^low^)

Next, to dissect the relationship between CD26 and the Akt-Snail/Slug pathway modulated by API, we first overexpressed Snail or Slug in A549 cells. Figure 5a shows that overexpression of Snail or Slug increased Akt activation, but had no significant effect on CD26 expression. Figure 5b further shows that the ectopic expression of myr-Akt significantly reversed the API-mediated inhibition of Akt activation, but not inhibition of CD26 expression. These results suggest that the Akt-Snail/Slug pathway does not modulate CD26 expression in API-treated NSCLC cells. In contrast, to determine whether CD26 modulates Akt activity and the Snail/Slug-mediated EMT, we knocked-down CD26 with CD26-specific shRNA in H1975 and A549 cells. Figure 5c shows that CD26 knockdown significantly decreased Akt activation and Snail, Slug, and fibronectin expressions, and the knockdown efficiency of CD26-specific shRNA was also shown here. Similar inhibitory effects of CD26 shRNA on Akt activation and Snail/Slug expression were also observed in CL1–5 cells (Additional file 1: Figure S3). From TCGA database, we observed that patients with CD26^high^/Akt^high^ lung tumors had the shortest recurrence-free survival times compared to the CD26^high^/Akt^low^, CD26^low^/Akt^high^, and CD26^low^/Akt^low^ groups (Fig. 5d). Taken together, these results revealed that CD26 may be vital in mediating the Akt-Snail/Slug-induced EMT in NSCLC cells, and API can target this pathway. Clinical data indicated that upregulation of CD26 and Akt may be critical events in promoting lung cancer progression.

### Significant anticancer effects of API via targeting CD26 in an A549 orthotopic graft model

**Fig. 6:**
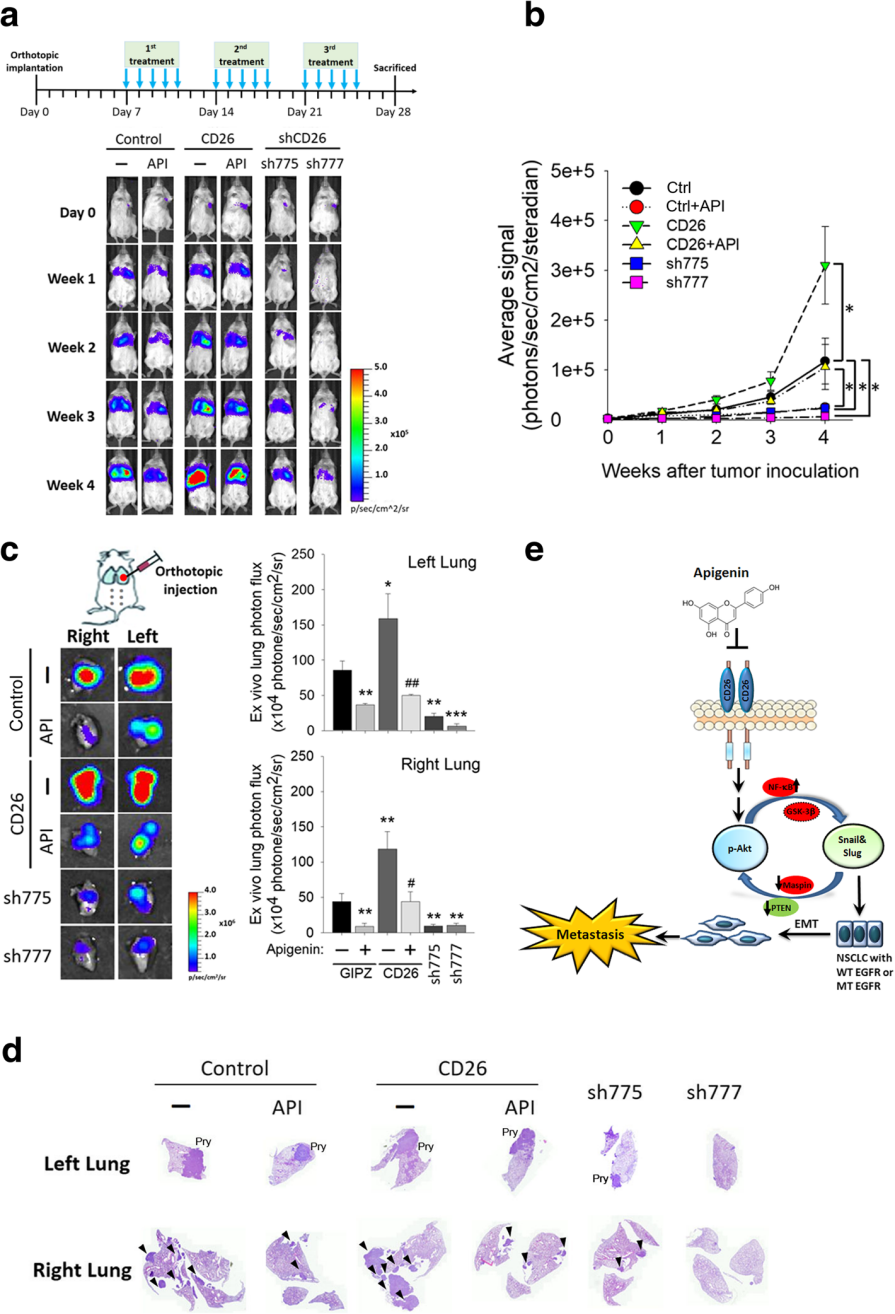
Targeting CD26 suppresses non-small cell lung cancer (NSCLC) growth and metastasis by apigenin (API) in an orthotopic mouse model. **a**, upper panel, Timeline of the in vivo study design for investigating the effects of CD26 expression on tumor progression and the antitumor activity of API. Male NOD/SCID mice were orthotopically injected with luciferase-tagged and CD26-overexpressing or CD26-depleted (sh-775 and sh-777) A549 cells. After 7 days, mice were treated with API (3 mg/kg, IP) or the vehicle for 5 days/week. **a**, lower panel, All mice were sacrificed at 4 weeks after API treatment, and the luciferase activity was detected every week with a noninvasive imaging system (IVIS imaging system, Xenogen). **b** Quantitative analysis of Xenogen imaging signal intensity (photons/s/cm^2^/steradian) every week. * *p* < 0.05. **c** Lungs were isolated and examined at the end of this spontaneous metastasis assay. Left panel: Cancer metastasis from the left lung to the right lung was imaged with bioluminescence at the end of the study. Right panel: Signal intensities from primary tumors (left lung) and metastatic tumors (right lung) were bioluminescently captured at the end of the study, with the mean signal for each group indicated. * *p* < 0.05, ** *p* < 0.01, *** *p* < 0.001 compared to the control group. ^#^ *p* < 0.05, ^##^ *p* < 0.01, compared to the API-treated control group. **d** H&E staining of lungs isolated from each group of mice; metastatic colonies are indicated by an arrow in the right lungs. **e** A working model shows the molecular mechanism underlying the ability of API to suppress the metastasis of NSCLC cells. The antimetastatic activity of API on NSCLC cells with wild-type or mutant epidermal growth factor receptor (EGFR) was attributed to inhibition of CD26 expression following suppression of the interplay between Akt activation and Snail/Slug expression, with ultimate restraint of EMT progression. Red ovals indicate hypothetical regulators which might be involved in the API-mediated interplay of Akt and Snail family members

To investigate the role of CD26 in tumor progression and in API-mediated anticancer activity in vivo, we established an orthotopic lung tumor-bearing model by transplanting luciferase-tagged cells, A549-mock-luciferase, A549-CD26-luciferase, or A549-shCD26-luciferase (sh-775 and sh-777), into NOD-SCID mice and allowed them to become established for 7 days before initiating treatment. A549–1-Luc orthotopic graft mice were IP-administered API or the vehicle 5 days/week. The effects of API administration or CD26 knockdown and overexpression on tumor growth and metastasis were monitored by bioluminescence imaging. A schematic timeline of this experimental design and setup is shown in upper panel of Fig. 6a. In vivo photon emission detection revealed that both API treatment and CD26 knockdown in A549 cells attenuated tumor growth compared to the control group (Fig. 6a, b). The tumorigenic ability was promoted in CD26-overexpressing A549 cells compared to control A549 cells and significantly reversed the antitumor growth effect of API (Fig. 6a, b). Similar to the in vivo data, after the mice were sacrificed, ex vivo imaging of their lungs respectively showed lower and higher photon intensities in A549/shCD26- and A549/CD26-injected mice compared to control mice, in both orthotopic tumors (left lung) and metastatic tumors (right lung) (Fig. 6c). The growth- and metastasis-inhibitory effects of API were also reversed in cancer cells overexpressing CD26 (Fig. 6c). Moreover, histological data also confirmed that mice bearing A549/shCD26 tumors or receiving API treatment all developed fewer metastatic nodules (as indicated by a black arrow) in the right lung compared to control mice (Fig. 6d). Taken together, these results revealed that API treatment suppressed the in vivo metastasis of NSCLC through targeting CD26.

## Discussion

Dissemination of cancer cells and resistance to drug treatment are two main causes for the poor prognosis of lung cancer patients. Cancer cells undergoing the EMT were reported to have increased resistance to targeted therapy and chemotherapy, higher invasive abilities, and acquisition of stem cell features [42]. Recently, API was used as a traditional medicine for its anticancer activities and low toxicity to normal cells. In both in vitro and in vivo models, API was shown to suppress tumor growth and metastasis in various cancer types. The antiproliferative activities involve inducing apoptosis and autophagy, modulating the cell cycle and stemness, stimulating an immune response, and enhancing chemosensitivity [29]. Moreover, API was also shown to inhibit migration, invasion, and metastasis of various cancer cells through suppressing the EMT or modulating ECM-degrading enzymes [29]. However, compared to other tumor types, little information on the effects of API on the migration and invasiveness of NSCLC cells is available. Until now, only one report indicated that API can inhibit the in vitro cell motility of NSCLC A549 cells by targeting Akt-mediated MMP-9 expression [30]. Our present results showed that API inhibited the migratory and invasive abilities of a series of NSCLC cell lines harboring WT (A549 and CL1–5) or different mutant EGFR statuses (HCC827 and H1975). Moreover, our results showed that API-mediated downregulation of MMP-2 and MMP-9 was only observed in A549 cells, but not in other NSCLC cell lines (CL1–5 and H1975), suggesting that inhibition of MMP-2 and MMP-9 by API might be cell-type specific. In contrast to MMPs, our study identified that CD26/DPPIV plays a critical role in regulating the invasive abilities of several NSCLC cell lines (A549, CL1–5, CL1–0, and H1975) and can be downregulated by API treatment in these cell lines. Moreover, the antimetastatic effect of API and the metastasis-promoting effect of CD26 were also observed in a human A549 xenograft model. These results suggested that suppression of CD26 might be a general phenomenon in API-regulated cell motility of NSCLC cells.

CD26 is a multifunctional cell-surface glycoprotein with intrinsic DPPIV activity, and it is widely expressed in most cell types. Cell-surface proteases participate in cancer progression and malignant transformation by facilitating tumor cell invasion and metastasis [43]. Clinical studies of urothelial carcinoma, thyroid cancer, colorectal cancer, and gastrointestinal stromal tumors indicated that CD26 expression is associated with distant metastasis or recurrence after resection [23, 25, 44]. Similar to previous findings, we also observed that lung cancer patients with a high CD26 expression level had significantly worse recurrence-free survival than those with a lower level. Pang et al. recently identified that CD26-positive colon cancer cells were associated with enhanced invasiveness and chemoresistance, which might be due to EMT induction [24]. Our results showed abundant CD26 expression in highly invasive NSCLC cell lines (A549, H1975, and CL1–5), but low CD26 expression in the poorly invasive CL1–0 cell line and found a significant correlation between CD26 protein levels and the invasive abilities of a set of NSCLC cell lines (A549, CL1–0, CL1–3, CL1–5, HCC827, PC9, and H1975). A similar correlation between CD26 expression and invasive abilities was also reported in T-cell malignancies [45]. Moreover, CD26 knockdown in NSCLC cells caused decreases in the invasive abilities and EMT-related markers (Snail, Slug, and fibronectin). Based on these results, we suggest that the CD26 level is a potential marker for predicting the invasive ability of NSCLC cells and the prognosis of lung cancer patients, and CD26 may regulate invasion of cells through EMT induction.

According to the oncogenic role of CD26 in cancers, CD26 itself appears to be a novel therapeutic target. For example, an anti-CD26 monoclonal antibody or CD26 inhibitor treatment was shown to inhibit growth and invasiveness against several tumor types such as renal cell carcinoma and colon cancer [26, 46]. Recently, several flavonoids, particularly luteolin, API, and resveratrol, were shown to inhibit the in vitro enzyme activity of CD26 [47]. Our present study further demonstrated that API also suppressed the protein and mRNA expressions of CD26 and the EMT-mediated cell invasion in several NSCLC cell lines (A549, CL1–5, and H1975). These results suggested that inhibition of CD26 might be a general phenomenon in API-regulated cell motility of NSCLC cells. Moreover, our studies also showed that API treatment attenuated growth and metastasis via targeting CD26 in an A549 orthotopic graft model. Taken together, our data suggest that the ability of API to inhibit cell invasiveness might be attributable to its capacity to suppress CD26 expression followed by inhibiting the EMT, and API has potential value in clinical applications for advanced NSCLC. Furthermore, as CD26 was reported to be a possible stem cell marker that induced the EMT in colon cancer [24], the role of CD26 and the effect of API on the stemness of NSCLC will be further investigated in the future.

Transcription factors of the Snail family (Snail and Slug) were associated with EMT progression during lung cancer metastasis [42]. Our results showed that protein levels of Snail family members exhibited dramatic decreases after API treatment of NSCLC cells. PI3K/Akt was reported to play a crucial role in regulating expressions of Snail family members through multiple mechanisms. For example, activation of PI3K/Akt can phosphorylate GSK-3β to promote GSK-3β degradation and further maintain the stability of Snail and Slug [14]. Moreover, activation of Akt can induce an increase of nuclear factor (NF)-κB subunit p65, further leading to increased Snail transcription [48]. Our present study demonstrated that API treatment could suppress Akt activation in several NSCLC cell lines (A549, CL1–5, and H1975). Moreover, overexpressing activated Akt in A549 cells induced upregulation of Snail and Slug and reversed API-mediated inhibition of the invasive ability, suggesting that Akt inhibition by API is a general phenomenon in NSCLC cells and may be the main cause for the API-mediated suppression of Snail family-induced cell motility. However, the effects of API on GSK-3β phosphorylation and p65 expression in NSCLC cells need to be further investigated in the future. Moreover, Akt activation was recently defined as a convergent feature of acquired EGFR TKI resistance, and increased p-Akt levels were observed in clinical specimens obtained from EGFR-mutant NSCLC patients who acquired EGFR TKI resistance [49, 50]. Combined treatment of Akt inhibitor with EGFR TKI had shown to exhibit the synergistic growth inhibitory effect in several EGFR TKI-resistant NSCLC models [49]. From the inhibitory effect of API on invasion and colony formation of NSCLC cells, our data also showed that EGFR mutant cells, HCC827 and H1975, were more sensitive to API treatment compared to the EGFR WT cells, A549 and CL1–5. Among these NSCLC cells, TKI-resistant H1975 cell was the most sensitive cells to API treatment. In addition to Akt, our study further demonstrated that API also suppressed the expression of p-EGFR in EGFR mutant cells (Additional file 1: Figure S4), suggesting the combination of API and EGFR TKI for the treatment of TKI-resistant NSCLC cells is worthy of further investigation.

As to upstream factors of PI3K/Akt signaling in cancer cells with EMT progression, transforming growth factor (TGF)-β was reported to act as an important inducer to stimulate the Akt-mediated EMT. TGF-β was reported to activate Akt directly or through upregulating hyaluronan synthase (HAS) expression, promoting CD44/EGFR expression and co-localization, and subsequently activating Akt in NSCLC cells [14, 51]. The effect of API on TGF-β expression and its downstream signaling molecules will be further investigated in NSCLC cells. In addition to TGF-β, the present study showed that knockdown of CD26 also suppressed Akt activation in NSCLC cells, suggesting that CD26 suppression by API might contribute to API-mediated inhibition of the Akt-induced EMT in NSCLC cells. Recent studies indicated that expression of CD26 in T cell lines leads to increased stromal-cell-derived factor (SDF)-1-α-mediated invasion through inducing PI-3 K/Akt pathways. Moreover, CD45 was reported to be associated with CD26 to regulate CD26’s enhancement of invasion [45]. Hence, the roles of SDF-1-α and CD45 in CD26-mediated invasive abilities of NSCLC and the effect of API on SDF-1-α and CD45 warrant further study in our future work.

In contrast to Akt-regulated expressions of Snail family members, our results also showed that overexpressing Snail or Slug in NSCLC cells could induce activation of Akt and reverse the API-mediated inhibition of Akt, suggesting that Snail family members showed positive feedback regulation of Akt activation. Previous studies reported that Snail or Slug can regulate Akt activation to induce prostate cancer cell motility and drug resistance through transcriptional inhibition of several tumor suppressors which target Akt, including maspin [52] and PTEN [53, 54]. In greater detail, Snail was reported to cooperate with lysine-specific demethylase 1 (LSD1) to repress PTEN through removing histone H3 lysine 4 [55]. Maspin and PTEN were shown to suppress survival of lung cancer cells through modulating the Akt pathway [56, 57]. Actually, our present and previous studies showed that Snail or Slug overexpression in A549 cells decreased maspin or PTEN expression [58], but the functional roles of maspin, PTEN, and LSD1 in Snail/Slug-regulated Akt activity and the effects of API on these regulators in NSCLC cells with different EGFR statuses should be further evaluated in the future.

## Conclusion

The present data demonstrated that API treatment of NSCLC cells which harbor the WT or mutant EGFR can suppress CD26 expression and the interplay of downstream targets, p-Akt and Snail/Slug, resulting in inhibition of the EMT-mediated invasive ability; the mechanism is schematically illustrated in Fig. 6e. Moreover, our study showed that increased CD26 expression was correlated with worse prognoses in lung cancer patients and higher invasive abilities of NSCLC cells. Furthermore, we also demonstrated the antimetastatic effect of API and the metastasis-promoting effect of CD26 in a human A549 xenograft model. Our present findings strongly support API being used as a potential preventive/therapeutic agent for managing NSCLC, and CD26 could be a useful biomarker for predicting NSCLC progression.

## Additional file

Additional file 1: **Figure S1.** Effect of apigenin (API) on colony formation in CL1–5 and H1975 non-small cell lung cancer (NSCLC) cells. **Figure S2.** Effects of apigenin (API) on changes in different proteases in A549 and H1975 cells following 24-h treatment with API. **Figure S3.** Knockdown of CD26 suppresses Akt activation and the epithelial-to-mesenchymal transition (EMT) in CL1–5 cells. **Figure S4.** Apigenin (API) inhibits phosphorylation of the epidermal growth factor receptor (EGFR) in EGFR-mutant HCC827 and H1975 cells. (DOCX 431 kb)

## Abbreviations

API: Apigenin
DPPIV: Dipeptidyl peptidase IV
ECM: Extracellular matrix
EGFR: Epidermal growth factor receptor
EMT: Epithelial-to-mesenchymal transition
GSK-3: Glycogen synthase kinase 3
MMP: Matrix metalloproteinase
NSCLC: Non-small cell lung cancer
PI3K: Phosphatidylinositol 3-kinase
PTEN: Phosphatase and tensin homologue
TKI: Tyrosine kinase inhibitor
WT: Wild-type
