# Supplementary material for: Downregulating CD26/DPPIV by apigenin modulates the interplay between Akt and Snail/Slug signaling to restrain metastasis of lung cancer with multiple EGFR statuses
Source: J Exp Clin Cancer Res. 2018 Aug 22;37:199. doi: 10.1186/s13046-018-0869-1 (PMC6104010; doi:10.1186/s13046-018-0869-1)
Supplement: Supplementary file 1 — Figure S1. Effect of apigenin (API) on colony formation in CL1–5 and H1975 non-small cell lung cancer (NSCLC) cells. Figure S2. Effects of apigenin (API) on changes in different proteases in A549 and H1975 cells following 24-h treatment with API. Figure S3. Knockdown of CD26 suppresses Akt activation and the epithelial-to-mesenchymal transition (EMT) in CL1–5 cells. Figure S4. Apigenin (API) inhibits phosphorylation of the epidermal growth factor receptor (EGFR) in EGFR-mutant HCC827 and H1975 cells. (DOCX 431 kb) [file 13046_2018_869_MOESM1_ESM.docx]

**Additional file 1**

**Title:**

**Downregulating CD26/DPPIV by apigenin modulates the interplay between Akt and Snail/Slug signaling to restrain metastasis of lung cancer with multiple EGFR statuses**

Jer-Hwa Chang, Chao-Wen Cheng, Yi-Chieh Yang, Wan-Shen Chen, Wen-Yueh Hung, Jyh-Ming Chow, Pai-Sheng Chen, Michael Hsiao, Wei-Jiunn Lee and Ming-Hsien Chien

Correspondence to: Dr. Wei-Jiunn Lee (E-mail: lwj5905@gmail.com) and Dr. Ming-Hsien Chien (E-mail: mhchien1976@gmail.com)

**Figure Legends**

**
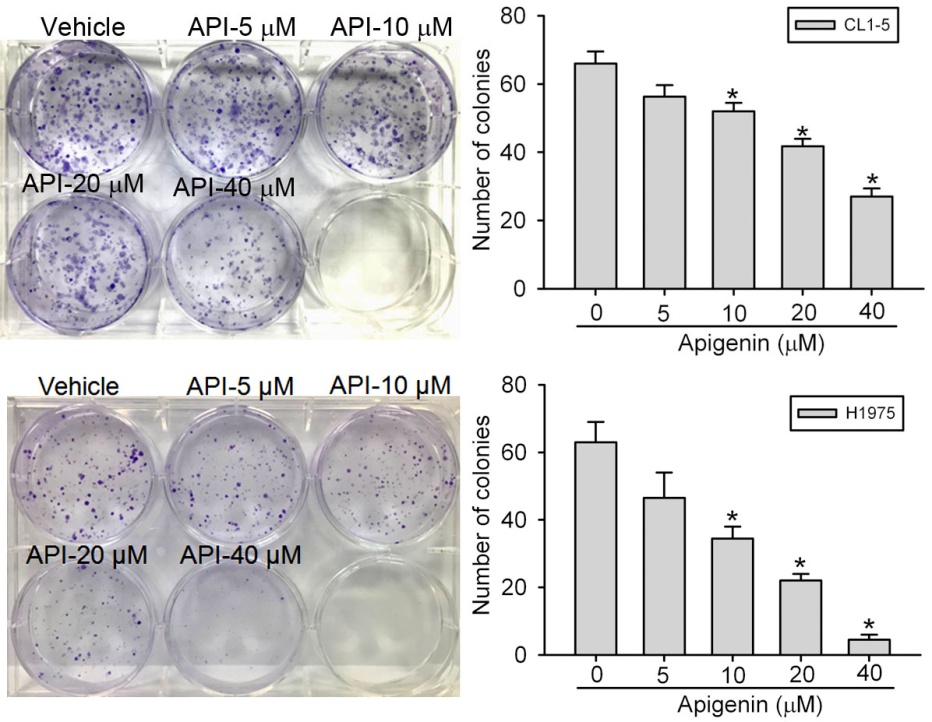
**

**Figure S1.** Effect of apigenin (API) on colony formation in CL1-5 and H1975 non-small cell lung cancer (NSCLC) cells. Cells were treated with vehicle or API (5~40 μM) for 24 h; then, the death-inducing effects of API on cells were determined by counting the colonies formed. Left: Representative photomicrographs. Right: Data are presented as the mean ± SD of at three independent experiments. * *p* < 0.05, compared to the vehicle group.


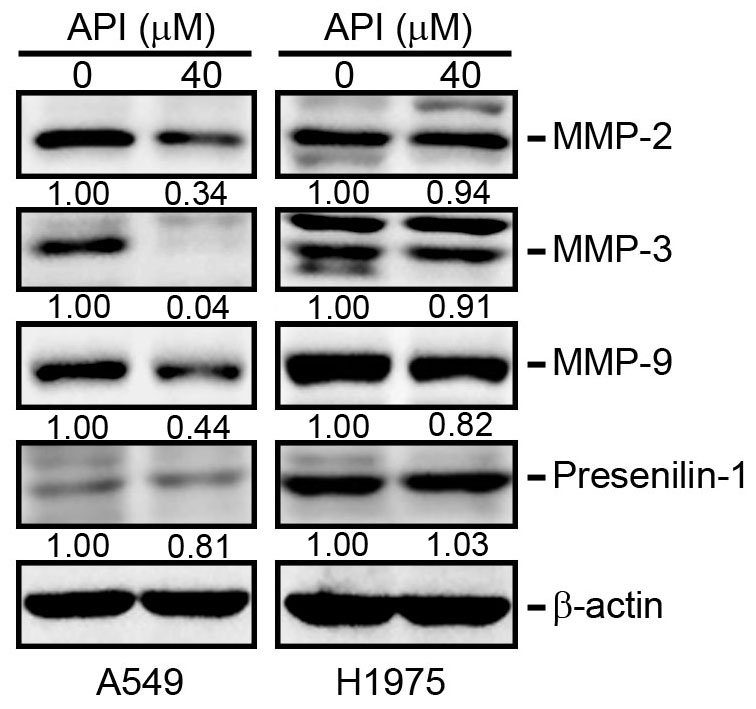


**Figure S2.** Effects of apigenin (API) on changes in different proteases in A549 and H1975 cells following 24-h treatment with API. Cells were harvested and lysed for the detection of matrix metalloproteinase (MMP)-2, MMP-3, MMP-9, presenilin-1, and β-actin by a Western blot analysis. Quantitative results of maspin protein levels, which were adjusted to the β-actin protein levels.

**

**

**Figure S3.** Knockdown of CD26 suppresses Akt activation and the epithelial-to-mesenchymal transition (EMT) in CL1-5 cells. CL1-5 cells were infected with a lentivirus carrying CD26 shRNA or shGFP (shCtrl) and subjected to a Western blot analysis to determine expressions of CD26, p-Akt, and EMT-related regulators (Snail and Slug) Quantitative results of p-Akt and other indicated proteins were respectively adjusted to total Akt protein and β-actin protein levels.


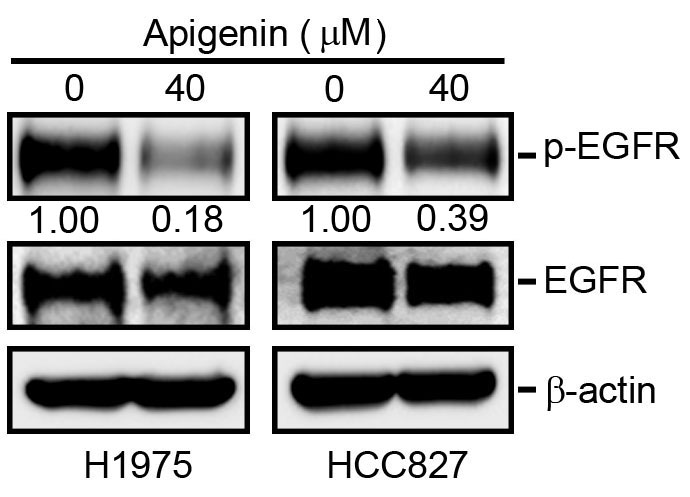


**Figure S4.** Apigenin (API) inhibits phosphorylation of the epidermal growth factor receptor (EGFR) in EGFR-mutant HCC827 and H1975 cells. Cells were treated with 40 µM API for 24 h and harvested for the detection of EGFR activation (phosphorylation) by a Western blot analysis. Quantitative results of p-EGFR levels, which were adjusted to the total EGFR protein levels.
